# Defects in Nitrogen-Doped ZnO Nanoparticles and Their Effect on Light-Emitting Diodes

**DOI:** 10.3390/nano14110977

**Published:** 2024-06-05

**Authors:** Raj Deep, Toshiyuki Yoshida, Yasuhisa Fujita

**Affiliations:** 1 Graduate School of Natural Science and Technology, Shimane University, 1060 Nishikawatsu, Matsue 690-8504, Japan; n21d103@matsu.shimane-u.ac.jp (R.D.); yosisi@riko.shimane-u.ac.jp (T.Y.); 2 S-Nanotech Co-Creation Co., Ltd., 1060 Nishikawatsu, Matsue 690-0823, Japan

**Keywords:** annealing atmosphere, defect luminescence, ZnO nanoparticles, p-type ZnO, light-emitting diode, nitrogen doping

## Abstract

In this study, the effect of defects on the acceptor properties of nitrogen-doped ZnO nanoparticles (NPs) was investigated through the fabrication of light-emitting diodes (LEDs). Nitrogen-doped ZnO NPs were synthesized by an arc discharge in-gas evaporation method and post-annealed at 800 °C in an oxygen and nitrogen atmosphere. The annealed ZnO NPs were characterized by X-ray diffraction, scanning electron microscopy, Raman spectroscopy, and photoluminescence spectroscopy. It was found that the annealing of nitrogen-doped ZnO NPs in a nitrogen environment increased the number of zinc vacancies, while annealing in an oxygen environment increased the number of oxygen vacancies due to nitrogen desorption. The output characteristics of LEDs fabricated with oxygen-annealed NPs were degraded, while those with nitrogen-annealed NPs were significantly improved. From these results, the contribution of zinc vacancies to acceptor formation in ZnO NPs was confirmed for the first time in actual pn junction devices.

## 1. Introduction

Semiconductor nanostructures can pave the way for nanophotonics and nanoelectronics in the domain of low-dimensional LEDs and other related devices. ZnO nanostructures have attracted intense interest as an important II-VI semiconductor due to their high exciton binding energy of 60 meV and direct and wide band gap in nature. Zinc oxide can not only be easily grown in a low-dimensional structure but can also be easily tailored with appropriate doping atoms, making it one of the most widely studied nanomaterials for a variety of applications such as photocatalysis [1,2], biomarkers [3,4], and optoelectronic devices, such as UV lasers [5,6], LEDs [7,8], and photodetectors [9,10].

Typically, ZnO nanostructures that are grown naturally exhibit n-type conductivity as a result of inherent defects, mostly oxygen vacancy (V_O_), zinc antisite (Zn_O_), zinc interstitial (Zn_i_), and/or hydrogen incorporation. In ZnO, the intrinsic donors’ self-compensation effect hinders p-type doping. Obtaining p-type doping in ZnO nanostructures has proven to be challenging and rarely accomplished due to the deep acceptor energy level and low solubility of the acceptor dopants. Acceptors should have a low formation enthalpy (ΔH) and must have shallow levels with respect to the valance band maximum. Zinc vacancy (V_Zn_), oxygen interstitial (O_i_), and oxygen antisite (O_Zn_) act as acceptor defects among the native defects in undoped ZnO [11]. In order to achieve optoelectronic devices with a high level of efficiency, it is imperative to have both high-quality p-type and n-type ZnO. Group III elements such as aluminum [12], gallium [13], and indium [14] are common choices as impurity dopants for enhancing the electron concentration in ZnO. P-type conduction was reported in ZnO nanowire arrays grown using group I elements, such as lithium [15] and sodium [16], and group V elements, such as antimony [17] and phosphorus [18], as dopants. However, the p-type conduction showed instability, which can be attributed to the larger atomic size of phosphorus or antimony when compared to oxygen atoms. In contrast, the smaller dopants occupy interstitial sites instead of substitution sites, where they act as donors [19]. Nitrogen is considered the primary acceptor dopant for ZnO among the group V elements because it has similar ionic radii and ionization energy to oxygen. However, the N substitution at the O site, N_O_, proved to be a deep acceptor. It might lead to higher ionization energy or a low hole concentration [20]. However, some theoretical findings also indicate the existence of shallow acceptors resulting from the presence of various complexes containing zinc vacancies and N such as N_O_-V_zn_ and N_Zn_-V_O_ [21,22]. Complexes with hydrogen, such as V_Zn_-N_O_-H^+^ and V_Zn_-H, may lower the low ionization energy and take part in shallow acceptor energy levels [21]. Thus, nitrogen-doped ZnO nanostructures are fabricated in various ways, leading to the acceptor nature of ZnO [23,24,25,26].

Moreover, it is widely recognized that the defect concentration in ZnO nanoparticles (NPs) has a significant impact on both their physical characteristics and optoelectronic properties. This defect concentration can be altered and regulated by adjusting the annealing conditions [27,28]. Generally, as-grown material with N dopants is likely to contain H-N complexes, and the annealing process may help to break them apart, thereby “activating” the N acceptors. Experiments have revealed that after high-temperature annealing, the majority of hydrogen-related local vibrational modes (LVMs) and multiphonon modes were no longer present [29]. Numerous experimental and theoretical discussions have been conducted on defects related to acceptors in zinc oxide. It has been noted that the presence of zinc vacancies theoretically promotes the incorporation of nitrogen acceptors. Nevertheless, there has been limited validation conducted using devices. Prior research has not verified the contribution of defects to the p-type nature of ZnO NPs through the fabrication of LEDs.

In this study, in order to understand the role of defects on the p-type properties of ZnO NPs, the characteristics of actual pn junction devices, LEDs, were evaluated to see how they are affected by defects. The contribution of zinc vacancies to the acceptor formation and the oxygen vacancies to the donor formation in the ZnO is studied and verified experimentally.

## 2. Materials and Methods

Nitrogen-doped p-type ZnO NPs were synthesized using an arc discharge apparatus (ULVAC Inc., Model No-GE-970, Chigasaki, Kanagawa, Japan). Prior documents provide a concise overview of the discharge process [30]. In this case, a zinc metal (purity 99.99%) was employed as the anode material. Within the arc discharge chamber, a zinc ingot was used as the anode, while a carbon rod was employed as the cathode. The chamber pressure of the arc discharge equipment was regulated via a control valve connected to a vacuum pump. A gas mixture consisting of nitrogen (N_2_) as the base gas with 19.9% oxygen (O_2_) was continuously introduced into the chamber at a flow rate of 5 L/min. After reaching the desired pressure, the arc was started with the carbon cathode and zinc anode positioned about 1 mm apart, resulting in the production of zinc plasma and gas plasma. Further, ZnO NPs were synthesized through the oxidation of zinc plasma using the vapor condensation method. In this study, a chamber pressure of 150 Torr and a current of 50 A were maintained to produce p-type ZnO NPs.

Thermal treatments were applied to the as-prepared ZnO NPs in the annealing furnace. In this study, two types of ambient gas were used: one was pure nitrogen gas with a purity of 99.99%, and the other was pure oxygen gas with a purity of 99.9%. After inserting the ZnO NPs in the chamber, the chamber’s air was removed by a vacuum pump, and pure gases were flushed to remove the contaminants. A constant gas flow of 0.5 L/min was supplied to the chamber during the whole process. In all instances, the temperature was elevated from the initial room temperature to 800 °C within a span of 10 min. Following a 60 min thermal treatment, the samples were subsequently cooled to a temperature below 40 °C within a period of two hours.

The size, morphology, and shape of the as-prepared ZnO and annealed NPs were examined using a field-emission scanning electron microscope, FE-SEM (JSM-7001FA, 5 kV, JEOL, Akishima, Tokyo, Japan). For the analysis of the crystal structure and phase purity of the ZnO NPs, X-ray diffraction was analyzed with CuKα radiation on a Rigaku Smart Lab diffractometer (Akishima, Tokyo, Japan). The intensity measurements were obtained within a 2θ range of 20–80°. For the optical properties of the NPs, the photoluminescence (PL) spectra of ZnO NPs were observed using a Horiba FluoroMax-4 spectrofluorometer (Minami-ku, Kyoto, Japan), using a 325 nm excitation wavelength emitted by a Xe lamp. The photoluminescence quantum yield (PLQY) of the as-prepared and annealed ZnO NPs is measured by using a Horiba F-3018 integrating sphere in a FluoroMax-4 spectrofluorometer (Horiba, Minami-ku, Kyoto, Japan).

**Figure 1:**
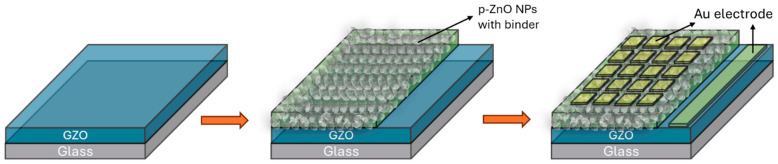
Schematics of the LED fabrication.

The Raman spectra of the NPs were additionally obtained utilizing the 532 nm laser line of a high-resolution Raman confocal system (Nanofinder 30, Tokyo Instruments, Edogawa-ku, Tokyo, Japan). To study the effects of the annealing on the ZnO NP-based LEDs, the LEDs were fabricated by spin coating using as-prepared and annealed ZnO NP samples. The schematics of the LED fabrication steps are shown in Figure 1. The dispersion of ZnO NPs was prepared by combining 0.05 g of NPs with 0.3 mL of isopropyl alcohol (IPA) and 0.1g of binder (Silsesquioxane OX-SQ SI 20; Toagosei Co., Ltd., Minato-ku, Tokyo, Japan). The dispersion was spin coated over the Ga-doped ZnO (GZO) thin film. The GZO films with an approximate thickness of 500 nm and a resistivity of around 4 × 10^−4^ Ω cm were deposited at 300 °C in an RF magnetron sputtering (Canon Anelva Corporation Model-400S, Kawasaki, Kanagawa, Japan) system using a 5% gallium-doped zinc oxide target. The spin-coating procedure commenced with an initial rotation speed of 1000 rpm for 5 s, then ramped up to 4000 rpm for 10 s. Following this, the coated layer of NPs underwent sintering on a hot plate set at around 300 °C for 1 min. The p-type layer is formed over an n-type thin film, forming a homojunction LED. Gold (Au) with a thickness of 30 nm was thermally evaporated for the p and n-type contact, with the area of the LED being 1 mm^2^. After the LED fabrication, electrical parameters such as the current–voltage measurements of LEDs were conducted using a semiconductor parameter analyzer, Keysight Technologies, B2900A, high-resolution SMU module (Hachioji, Tokyo, Japan). The electroluminescence (EL) spectra from the corresponding LEDs were evaluated at room temperature by measuring from the top side of the LED, p-contact Au electrode, utilizing the Ocean Optics QE65000 fiber multichannel monochrome meter. The output EL power of the LEDs was determined by positioning Si-based photodiodes (S2281, Hamamatsu Photonics, Higashiku, Hamamatsu city, Japan) beneath the LEDs.

## 3. Results and Discussion

**Figure 2:**
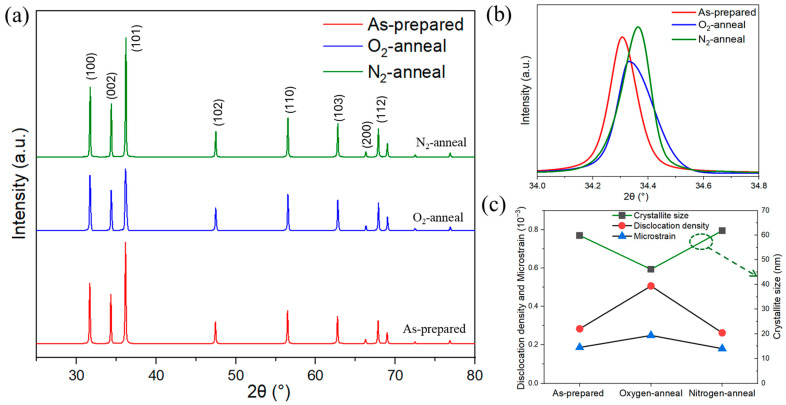
(**a**) X-ray diffraction pattern of as-prepared and annealed ZnO NPs, (**b**) XRD peak of the (002) plane, (**c**) relation between dislocation density, microstrain, and crystallite size.

The XRD pattern confirmed the wurtzite crystal structure and highly crystalline nature of the as-prepared ZnO and annealed NPs, which agreed with the diffraction peaks, following the reported Joint Committee on Powder Diffraction Standards (JCPDS), card number 36–1451. The absence of additional diffraction peaks corresponding to other binary compounds suggests that the ZnO NPs synthesized using the DC arc discharge method have a high level of crystallization quality. In the as-prepared ZnO NPs and annealed ZnO NPs, the orientations of the crystallites were favored along the (100), (002), and (101) planes. After annealing the samples at 800 °C in an O_2_ and N_2_ environment, the overall diffraction pattern remained similar, as shown in Figure 2a. When ZnO NPs were annealed in the N_2_ environment, the peaks shifted to higher angles, whereas in the O_2_ environment, the broadening of diffraction peaks was observed with a reduction in the peak intensity, as shown in Figure 2b, which represents the XRD peak of the (002) plane. The broadening of the diffraction peaks is influenced by both the size of the crystallite and the microstructure of the lattice. Nonetheless, detailed analysis of the XRD data reveals the structural differences between the annealed particles, such as the crystallite size and dislocation density, as well as the microstrain.

The average size of the crystals in the samples was determined using the Debye–Scherrer equation, which involved analyzing the diffraction intensities in the (100), (002), and (101) planes. Analysis of the XRD data using the equation for annealed and as-prepared ZnO NPs revealed that the average crystallite size decreased from 59 nm to 46 nm when annealed in an O_2_ environment, while the crystallite size increased to 61 nm when annealed in a N_2_ environment, as shown in Figure 2c.

The crystallite size can be calculated by $D=\frac{0.9\lambda }{\beta \cos\theta }$, where λ represents the wavelength of the radiation, θ represents the Bragg angle, and β represents the full width at half maximum (FWHM) of the diffraction peaks. Estimating the size of crystallites also enables the assessment of the structural integrity of the polycrystalline NPs by calculating the parameter δ = (1/D^2^), which has been found to be linked to the density of dislocations. The δ parameter provides a means to qualitatively assess structural imperfections such as grain boundaries, which have an impact on the crystallographic quality of NPs [31]. In the annealed O_2_ environment, the average dislocation density of the ZnO NPs increased, while in the N_2_ environment, it decreased when compared to the prepared ZnO NPs. The lower value of δ indicates that the NPs annealed in a nitrogen environment have a minimal number of lattice defects and exhibit superior crystalline qualities compared to the samples annealed in oxygen.

The microstrain (*η*) is estimated using the relation, $\eta =\frac{\beta }{4\tan\theta }$. As can be seen from Figure 2c, the microstrain in the ZnO NPs when annealed in the N_2_ atmosphere was similar to the as-prepared NPs. The comparison of the microstrain between the as-grown ZnO NPs and those annealed under the N_2_/O_2_ atmosphere reveals that the annealed ZnO in the O_2_ atmosphere experienced increased microstrain in comparison with the as-prepared ones, in accordance with the dislocation density.

**Figure 3:**
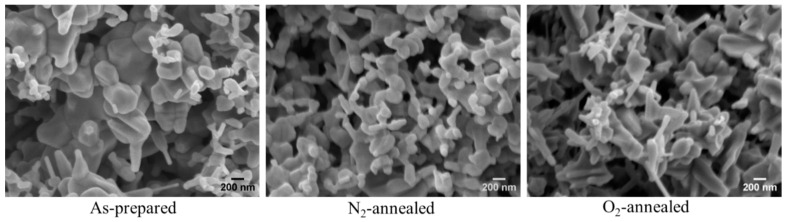
SEM images of as-prepared and annealed ZnO NPs.

Figure 3 displays the scanning electron microscope (SEM) images of ZnO NPs before and after annealing in an N_2_/O_2_ atmosphere. The scattering and cooling of plasma under non-equilibrium conditions resulted in the interaction of plasma components and the formation of clusters and NPs. The morphology of the ZnO NP samples, both the as-prepared and N_2_-annealed, exhibited similarities with a combination of nanorods, NPs, and tripods, displaying a well-defined crystal structure. However, in the crystal structure of the O_2_-annealed ZnO NPs, these characteristics were not as prominent in comparison, probably due to the increased microstrain or dislocation density.

**Figure 4:**
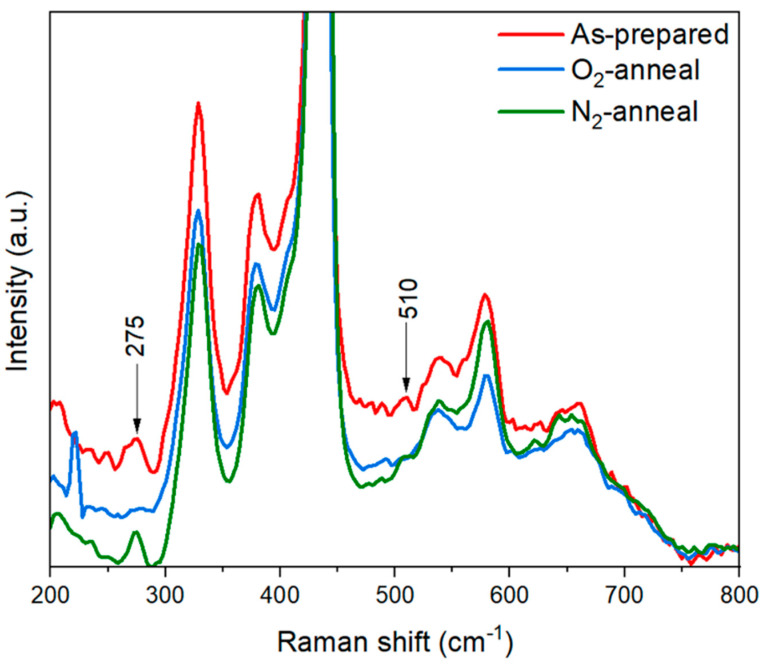
Raman spectra of as-prepared and annealed ZnO NPs.

The as-prepared ZnO NPs and annealed ZnO NPs were examined using a confocal micro-Raman spectrometer to investigate the impact of annealing on the inclusion of nitrogen in ZnO. The ZnO synthesized has a hexagonal wurtzite structure in correspondence to the P6_3_mc space group. Figure 4 shows the Raman spectra of the as-prepared and annealed ZnO NPs in ambient N_2_ and O_2_. The dominant Raman active non-polar phonon mode at 438 cm^−1^ (E_2_^high^), which is characteristic of the hexagonal wurtzite crystal structure, was observed in all samples. Other polar phonon modes, such as the A_1_ (TO) and E_1_ (LO) optical modes, appeared at 380 cm^−1^ and 584 cm^−1^, respectively. Raman peaks at around 330 cm^−1^ represent E_2_^high^ − E_2_^low^, peaks around 540 cm^−1^ represent 2B_1_^low^, and peaks around 650 cm^−1^ represents TA + LO [32]. Along with the intrinsic modes, some nitrogen-related local vibrational modes (LVMs) were also identified in the Raman spectra at 275 cm^−1^ and 510 cm^−1^ in the as-prepared samples. Here, the presence of nitrogen-related LVMs supported the acceptor nature of the p-ZnO NPs [33]. Nitrogen is probably doped in atomic form in ZnO NPs by replacing O crystal sites. Wang et al. discussed that the vibrational mode from Raman spectroscopy at 275 cm^−1^ is caused by a Zn atom in which some of its closest neighboring O atoms have been substituted with N atoms [34]. Friedrich et al. verified this experimentally and attributed this vibrational mode to the Zn_i_-N_O_ complex [35]. After annealing the samples in the O_2_ atmosphere, the nitrogen-related LVMs significantly diminished, while they were retained in the case of the N_2_-annealed samples.

**Figure 5:**
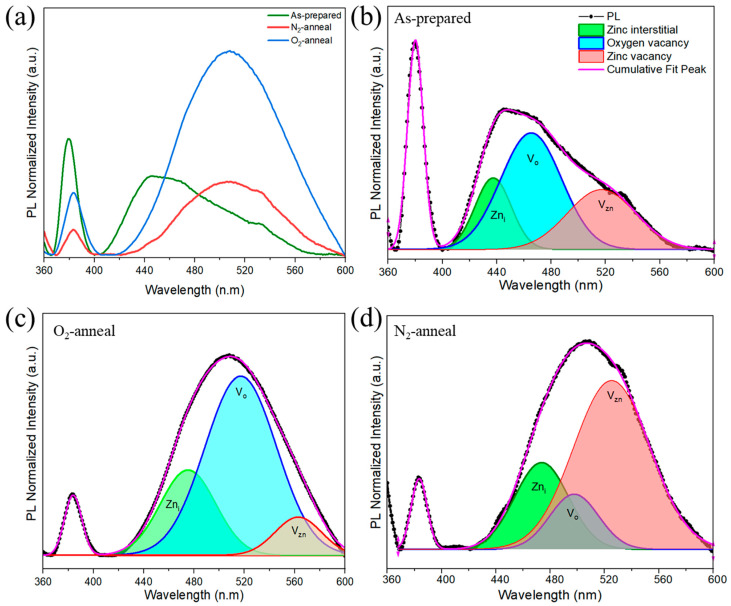
(**a**) PL spectrum of as-prepared and annealed ZnO NPs, (**b**–**d**) deconvoluted PL spectrum of as-prepared and annealed ZnO NPs.

The optical properties of the as-grown ZnO NPs were measured by PL spectrum at room temperature, as shown in Figure 5a. The PL was excited by weak-intensity UV light (Xenon lamp), which led to a spectrum consisting of two characteristic peaks, a sharp peak in the near UV region (380 nm), and a broad and deep-level emission peak in the visible region. Generally, exciton emission is seen at about 380 nm at room temperature for weakly excited PL. In the case of EL, it shifts to the longer wavelength side due to the higher excitation density and the heat generated by current injection [36]. The PLQY of as-prepared samples was around 3.5%. The PLQY increased to 4.6% in the case of nitrogen-annealed samples; meanwhile, it decreased to 3% for oxygen-annealed samples.

The UV intensity peak was weakened after annealing, while redshift was observed in the visible region for the annealed samples. Post-annealing frequently leads to a decrease in the strength of the near-band-edge (NBE) emission as a result of the contamination caused by the dopants. The reduction in the NBE emission can be attributed to changes in the intrinsic defects, such as V_Zn_, V_O_, and Zn_i_ [27]. To investigate the effects of the annealing ambience on the luminescent properties of ZnO NPs, the PL spectra were deconvoluted in four major distinct peaks using Gaussian functions: the UV emission at 383 (±3) nm, Zn_i_ at 473 (±3) nm, V_o_ at 497 (±3) nm, and V_zn_ at 523 (±3) nm, respectively. The deconvoluted PL spectra for the as-prepared ZnO NPs are shown in Figure 5b, the O_2_-annealed ZnO NPs are shown in Figure 5c, and the N_2_-annealed ZnO NPs are shown in Figure 5d. In the case of the O_2_-annealed samples, there was an increase in oxygen-related vacancies, which are responsible for donor-related properties. Meanwhile, in the N_2_-annealed samples, V_zn_ was dominant, indicating better acceptor properties [37]. After high-temp annealing, V_O_ tends to increase in an oxygen environment, while it is weakened in a nitrogen environment. The increase in V_O_ might be due to the out diffusion of nitrogen. The nitrogen concentration for the prepared samples was in the range of 10^18^~10^19^ cm^−3^ as measured by thermal conductivity methods [38]. This concentration included surface adsorption, so the nitrogen displacing oxygen sites will be different. Annealing reduces it to about 1/10 of the value. The Raman results also support the fact that nitrogen is being removed from the oxygen sites when annealed in ambient oxygen. For the case of undoped ZnO nanorods, the oxygen vacancy decreased with the annealing in an increased oxygen partial pressure environment [39].

**Figure 6:**
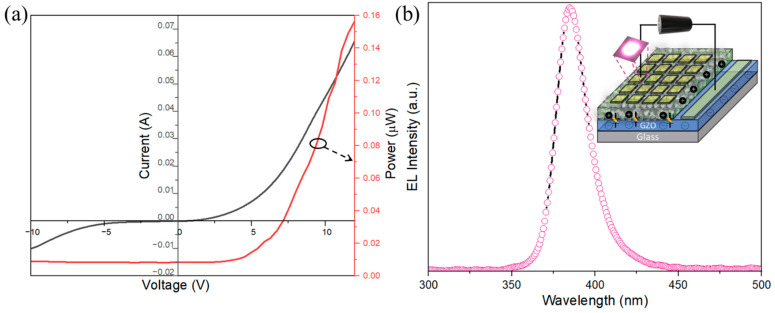
(**a**) *I–V* and power output of the fabricated LEDs using as-prepared ZnO NPs, (**b**) EL spectra of ZnO NPs, inset: UV-LED schematics.

**Figure 7:**
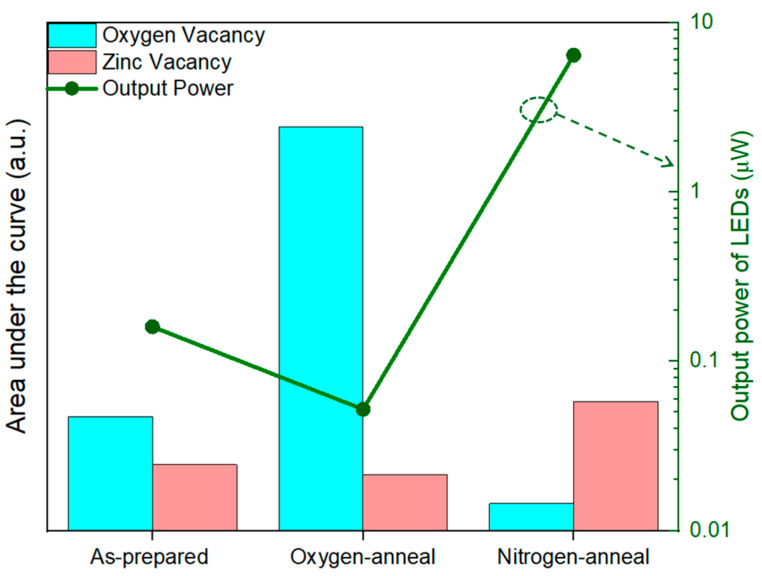
Relation between defect luminescence and output power of LEDs.

To ascertain the acceptor-related properties, LEDs were fabricated using as-prepared and annealed p-type ZnO NPs. Figure 6a shows the *I–V* results and power output of the fabricated LEDs using as-prepared ZnO NPs. The *I–V* characteristics reveal a nonlinear curve with a diode-like rectification character. The threshold voltage of the LEDs was close to the band gap of ZnO, i.e., 3.5 V at room temperature. The p-type ZnO and n-type ZnO layers exhibited favorable ohmic behavior when they came into contact, as demonstrated in prior research [40]. LEDs show high leakage current in the forward- and reverse-bias regimes because of the binder, which thereby increases the resistivity of the p-type layer. The output of the light emission was measured by the photodiode kept beneath the LEDs. It should be noted that the LEDs’ luminous output was measured from one side; hence, the light transmitted by total reflection on the glass substrate was not taken into consideration. Thus, the output power of the LEDs was used for relative comparison. The actual output of the LEDs was estimated to be 12 times higher than the measured output power, caused by the absorption and reflection within the glass substrate [41]. Figure 6b displays the electroluminescence (EL) spectra of the LEDs when a forward bias voltage of 10 V is applied. The EL spectra exhibited UV emissions exclusively near the band edge, while emissions related to the defects became saturated due to the increased injection of the current during EL, which was present during PL when excited by weak-intensity light [36]. The observations revealed narrow electroluminescent spectra with an FWHM of 172 meV, peaking at 383 nm. This suggests that the emission was a result of the radiative annihilation of excitons. Figure 6b, inset, depicts the schematic diagram of the ZnO-NPs-based LED under forward bias. Meanwhile, the output power of the as-prepared and annealed ZnO NP-based LEDs was compared and was related to the defect luminescence, as shown in Figure 7. The LEDs with ZnO NPs annealed in N_2_ showed better output characteristics when compared with as-prepared samples and, conversely, the output characteristics of the LEDs were degraded when annealed in an O_2_ environment. In the case of the O_2_-annealed samples, the output of the LEDs suffered as oxygen vacancy-related defects were prevalent in the sample. The reduction in acceptor-like properties in the ZnO NPs was observed probably with the increase in the oxygen deficiency and the presence of relatively less nitrogen, which acts as an acceptor dopant. Thus, V_o_ can be attributed to introducing the n-type conductivity in ZnO, which was theoretically predicted and also experimentally verified [37], whereas an increase in zinc vacancy-related defects was dominant in the nitrogen-annealed samples, leading to the enhanced output power of the LEDs. The increased output power of the LEDs can be attributed to the effective radiative annihilation of excitons, which could be the result of an increase in acceptor-related defects. Thus, zinc deficiency could facilitate lowering the energy required for the acceptor level. Similar results have been experimentally verified by Chavillon et al. using electrochemical and transient spectroscopy, which concluded that the stability of p-type conductivity was due to Zn-poor NPs [25]. The increase in PLQY of ZnO NPs helps in the increase in carrier density, which leads to an increase in the activation rate of acceptors, thus improving EL. There is a clear indication that the main cause of the p type is the combined effect of acceptor doping and zinc deficiency in nitrogen-doped ZnO NPs, as predicted by theoretical calculations, which was further supported by experimental results using PL, Raman spectroscopy, and actual device formation. Although ZnO has attracted global attention, the practical implementation of devices has not been achieved due to the absence of high-quality p-type materials. This study successfully validated previous theoretical predictions using actual devices. This study also offers valuable understanding of the p-type conduction mechanism in ZnO and will aid in the practical implementation of ZnO-based light-emitting devices.

## 4. Conclusions

In summary, ZnO NPs-based LEDs were fabricated using the nitrogen-doped ZnO NPs produced by the arc discharge method and were used to study the acceptor formation principle of ZnO. The role of defects in nitrogen-doped ZnO NPs was studied and verified experimentally by fabricating the LEDs using NPs. The experimental results revealed that zinc vacancy-related defects in nitrogen-doped ZnO act as acceptors and facilitate enhancing the p-type properties of ZnO. In contrast, oxygen vacancy-related defects suppress the output of the fabricated LEDs, thereby acting as donors. Finally, this is the first experimental evidence that zinc vacancies improve the p-type characteristics of nitrogen-doped ZnO NPs. These findings are a significant advancement in identifying the main inherent and external defects that influence the acceptor-like properties of nitrogen-doped ZnO NPs. These findings open opportunities to understand and fabricate p-type ZnO-based photonic devices.

## Data Availability

Data are contained within the article.
